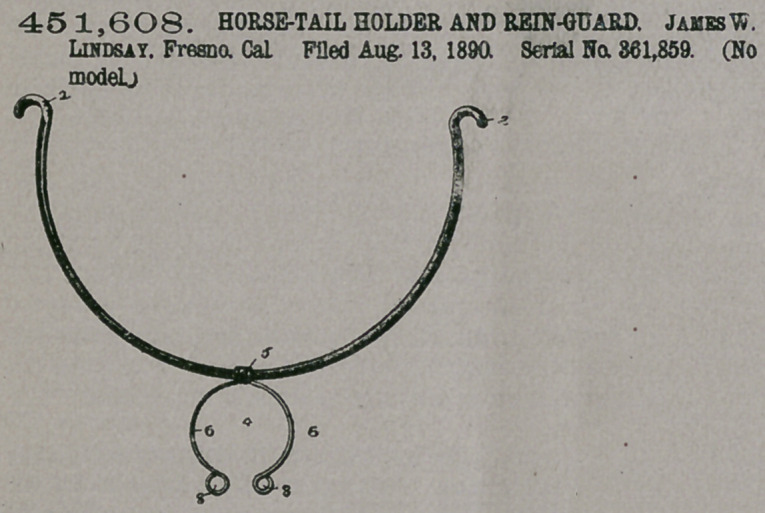# Recent Patents

**Published:** 1891-06

**Authors:** 


### RECENT PATENTS

RELATING TO

### VETERINARY MEDICINE AND ANIMAL INDUSTRY.

*Issued by U. S. Patent Office for Month ending May,* 1891.

**450,170. WATERING-TROUGH FOR STOCK.** WILLIAM L. MAGEE, Bethany, Mo. Filed Apr. 8, 1890. Serial No. 347,187. (No model.)

*Claim.*—A water-trough comprising the following parts in combination: a water-trough pivotally mounted at its forward end, a weighted lever connected with the rear end of said trough, the cylindrical tube G, connected with a source of water supply, a waterpipe extending from said tube toward said trough, an elbow-lever having its lower end connected with the rear end of said trough, a valve in said tube, and a rod connecting together the upper end of said elbow-lever and said valve.

**450,525. ANIMAL-POKE.** JOB F. PHILLIPS, Weakley County, Tenn. Filed Nov. 6, 1890. Serial No. 370,470. (No model.)

*Claim.*—1. In an animal-poke, the combination of the poke bar A, the upper end of which is bifurcated for the reception of a pivoted spur-wheel, and rigid or metallic check or side pieces B, secured at an upward inclined angle to the poke-bar and provided with openings *b b* and *c c* for the reception of straps C, D, and E, substantially as set forth.

2. In combination with a poke-bar A, pivoted spur-wheel *a*, and rigid cheek or side pieces B B, said cheek or side pieces having openings *b* and *c*, nose-strap C, throat-latch D, and strap E, having members *e e*1, said straps being adapted to retain the poke upon the head of an animal and limit its play, substantially as set forth.

**450,436. FOOT-RASP FOR HORSES.** GILBERT TOMPKINS, San Leandro, Cal. Filed July 17, 1890. Serial No. 359,072. (No model.)

*Claim.*—1. A foot-rasp comprising a bar having a rasp-surface shaped to cover the whole tread of the hoof, an apperture to clear the frog, and means for grasping and operating it arranged in the plane of the bar, as set forth.

2. A foot-rasp comprising a bar having a rasp-surface on one face, and a file surface on the other, shaped to cover the whole tread of the hoof, an apperture to clear the frog, and means for grasping and operating it arranged in the plane of the bar, as set forth.

**450,622. HORSESHOE.** CHARLES J. JUTSON and FREDERICK A. POUPARD, London, England. Filed Aug. 13, 1889. Serial No. 320,632. (No model.)

*Claim.*—1. The shoe proper having hooks at its respective heel ends and a toe-pillar in front and constructed with tread-studs in the form of ridges upon the upper tread, one of which is placed transversely immediately behind the toe-pillar, one radically on each flank, and two diagonally near the heel ends, these last-named tread-studs converging to a point at the front of the shoe, combination with side bands and connections interlocking with said hooks and with the upper end of said toe pillar, substantially as and for the purposes described.

2. In a nailless horseshoe, the combination of a toe-pillar provided with a spring at its inner base, with a tread-stud placed immediately behind the said pillar to effect the locking of the shoe upon the hoof, substantially as described.

3. In a nailless horseshoe, a toe-pillar constructed with a bifurcated upper end and with a pliable central prong located in front of the fork and adapted to be upset rearwardly into the intertine space of the fork, in combination with a fastening-band crossing said fork behind said central prong, substantially as described.

4. In combination with a fastening-band having eyes at its rear ends, the shoe proper constructed with laterally-projecting hooks at its heel ends to engage with said eyes, and projecting ridges which project laterally below the respective hooks, substantially as described and shown.

5. In combination with a fastening-band and suitable connections, and shoe proper constructed with tread-studs in the form of ridges located on the top tread near the heel ends and converging toward the front part of the shoe, substantially as described and shown, for the purpose set forth.

**450,650. HOPPLE.** HIRAM TWIST, Erie, Pa. Filed Aug. 30, 1886. Serial No. 212,223. (No model.)

*Claim.*—The combination of the leg-straps having the forward end secured to a loop, a neck-strap the ends of which are secured to a loop supporting a plate B, said plate B having a tongue or shackle B1, pivoted at one end to the plate, which is also provided with a catch-lever engaging with the free end of the tongue or shackle B1, and releasing-cord secured to said catch-lever.

**451,049. CATTLE-DEHORNER.** Asa W. Hindman, Chester, Nebr. Filed Jan. 20, 1891. Serial No. 378,434. (No model.)

*Claim.*—1. In a cattle-dehorner, the combination, the inner end of which terminates in a handle, the upper end of which is laterally curved and thickened to form an abutment, a pair of clamping-plates connecting the curved end with the member below its curved position, a yielding packing interposed between the two plates, and a series of adjusting-bolts passed through the plates and packing, of the cutting-member pivoted to the guiding member immediately below its curved end, a crescent-shaped knife extending from the cutting member at one end and riding at its outer end between the clamping-plates, and the inner end of the cutting member terminating in a handle, substantially as specified.

**450,826. TOE-WEIGHT FOR HORSES.** Henry E. Long, Des Moines, Iowa. Filed Aug. 30, 1890. Serial No. 363,535. (No model.)

*Claim.*—1. A toe-weight for horses, consisting of a clip adapted for permanent attachment to the hoof of the horse and having series of ratchet-teeth, a weight, and a spur adapted for attachment to the weight and having ratchet-teeth which mesh with those of the clip when the parts are adjusted, as and for the purposes stated,

2. A toe-weight for horses, consisting of a clip having embracing-arms on which are formed ratchet-teeth, which clip is adapted for permanent attachment to the hoof, a weight, and a spur removably secured to the weight, having ratchet-teeth which mesh with those of the clip when the parts are adjusted on the hoof, as and for the purposes described.

3. A toe-weight for horses, consisting of a clip having embracing-arms and ratchet-teeth formed on said arms and adapted for permanent attachment on the hoof, a weight, and a spur adapted to and held upon the weight by an adjusting-screw, said spur having ratchet-teeth which mesh with those of the clip when the parts are adjusted on the hoof, as and for the purposes set forth.

**450,758. ANIMAL-YOKE.** Joseph N. Montgomery, Lacon, Ill., assignor of one-half to Anthony Hacker, same place. Filed Dec. 18, 1889. Serial No. 334,231. (No model.)

*Claim.*—1. In an animal-poke, a neck-yoke or collar A, having a poke rod B pivotally secured between its lower extreme-tremeties by means of a pin or bolt, and a brace C, pivotally secured by a second pin immediately below the said poke-rod and between the said projections, substantially as and for the purposes set forth and described.

2. In an animal-poke, a neck-yoke or collar A, having a poke rod B pivotally secured between its lower extremities by means of a pin or bolt, and a brace C, pivotally secured thereunder by a pin, the rear or pivoted end of the poke-rod B extending slightly beyond the pivoted end of the said brace C, for the purpose herein set forth and described.

**451,655. VETERINARY INHALER.** HENRY T. WELCH, San Jose, Cal. Filed Sept. 5, 1890. Serial No. 364,075. (No model.)

*Claim.*—1. An inhaler for horses, consisting of a hollow medicine-receptacle having top perforations and bent to fit the upper lip of the horse below the nostrils, substantially as described.

2. An inhaler for horses, consisting of the hollow medicine-receptacle having screw-capped ends and top perforations, and suitable straps to hold said receptacle in place under the nostrils of horse, substantially as herein described.

3. An inhaler for horses, consisting of the curved hollow medicine receptacle having the top perforations, the cross-strap connected with said receptacle, and the front strap connected with said receptacle and with the cross-strap, substantially as herein described.

4. An inhaler for horses, consisting of the curved hollow receptacle provided with top perforations, the cross-strap and front strap fitted to said receptacle by sliding loops, whereby they can be removed, and the side straps for connecting the receptacle with the head-gear of the horse, substantially as herein described.

**451,647. ATTACHING TOE-WEIGHTS TO HORSESHOES.** GEORGE W. WEMPLE, Boston, Mass. Filed Sept. 20, 1890. Serial No. 365,642. (No model.)

*Claim.*—In combination with the main body of a horseshoe, the clip *a*, having its lower side about midway of the vertical thickness of said main body and provided with the oblique hole *b*, the lower end of which is enlarged, the toe-weight B, having the recess *c* in its inner face to receive the clip *a* and provided with the hole *d*, the bolt *e*, provided with the head *c*1, adapted to fit the enlarged portion of the hole *b*, and the nut *f*, all constructed, arranged, and operating substantially as described.

**451,332. HORSESHOE.** GEORGE D. LYNCH, Pittsfield, Ill., assignor of one-half to Willard G. James, same place. Filed Aug. 15, 1890. Serial No. 362,095. (No model.)

*Claim.*—1. In a horseshoe, the combination, with the shoe proper having apertured heel-calks, of a spring bowed or sprong beneath the quarters of the shoe and provided with end tenons fitting in the apertures of the calks, substantially as set forth.

2. In a horseshoe, the combination, with the shoe proper having apertured heel-calks, of a spring having its ends provided with tenons fitting in said apertures and also provided at its forward portion or toe upon opposite edges with upwardly-extending clamping-lugs, substantially as set forth.

3. In a horse-shoe, the combination, with the shoe proper having apertured heel-calks, and also havins its toe re-enforced or enlarged upon its under face, of a spring having its ends provided with tenons fitting in said apertures and provided at its forward portion or toe and upon opposite edges with upwardly-extending lugs adapted to clamp the re-enforced or enlarged portion of the toe of the shoe, said toe and the corresponding part of the spring being also provided with registering holes for the reception of nails, substantially as set forth.

451,608. HORSE-TAIL HOLDER AND REIN-GUARD. JAMES W. LINDSAY, Fresno, Cal. Filed Aug. 13, 1890. Serial No. 361,859. (No model.)

*Claim.*—1. The here-in-described rein-guard, consisting of a guard or support adapted for connection to the harness and of a tail-holder formed of spring-wire bent to form a spring-coil 5 for loosely receiving the guard, and at each side of the coil with curved arms 6, substantially as specified.

2. The bow-shaped guard 1, terminating in hooks or eyes 2, adapted to pass through perforations formed in the hip-straps of a harness, and the tail-holder, the same consisting of the central spring-coil 5 for loosely receiving the guard, and provided at each side of the coil with curved arms 6, diverged to form the tail-embracing portion and terminating in the eyes 8, substantially as specified.

## Figures and Tables

**Figure f1:**
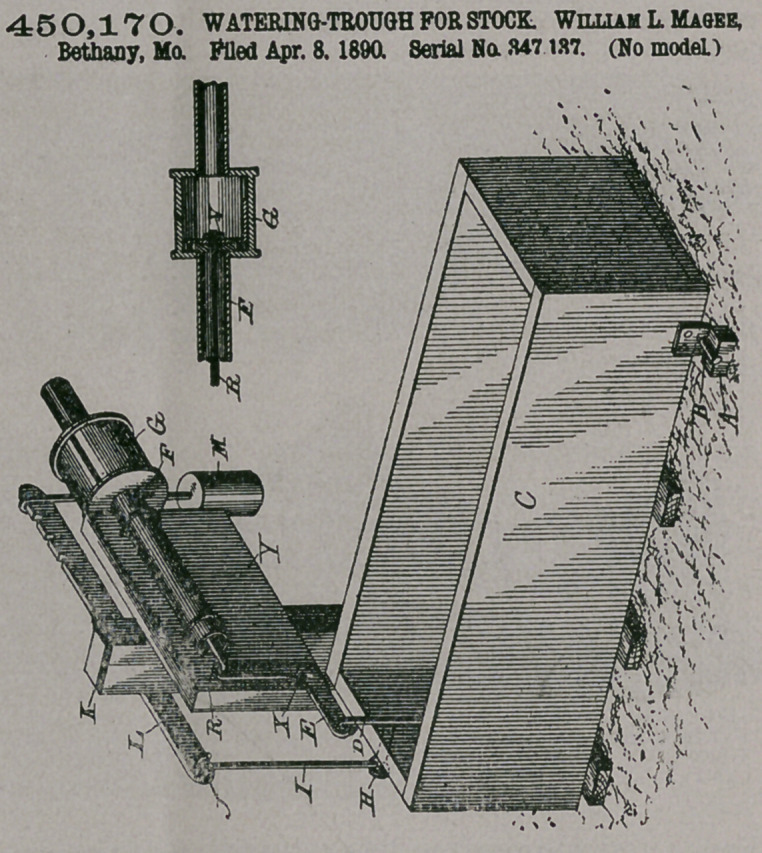


**Figure f2:**
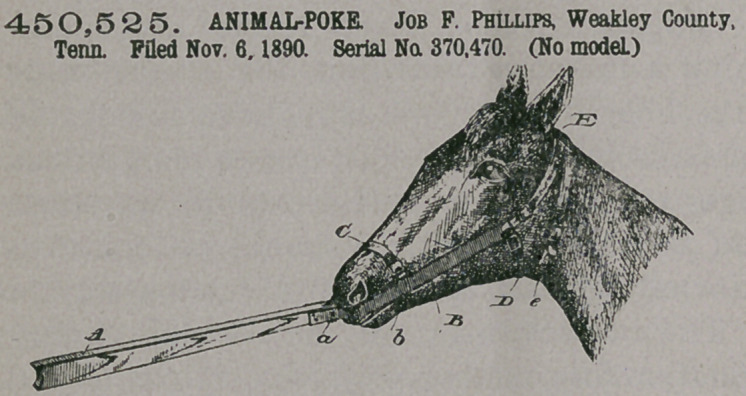


**Figure f3:**
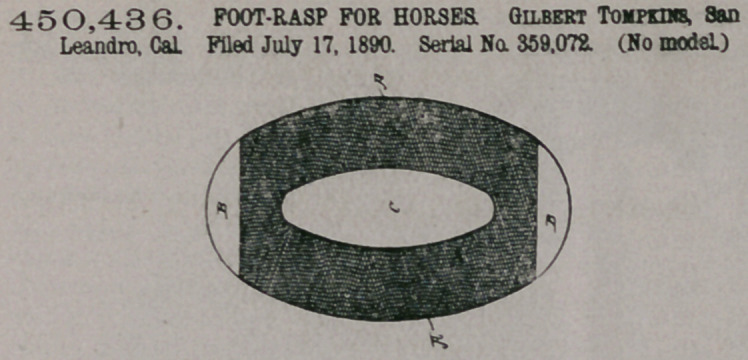


**Figure f4:**
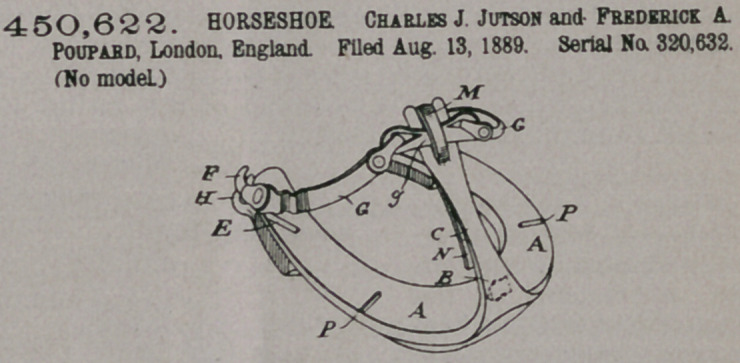


**Figure f5:**
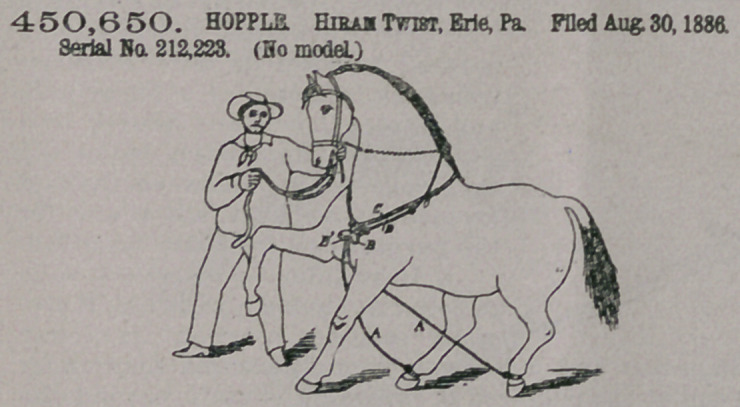


**Figure f6:**
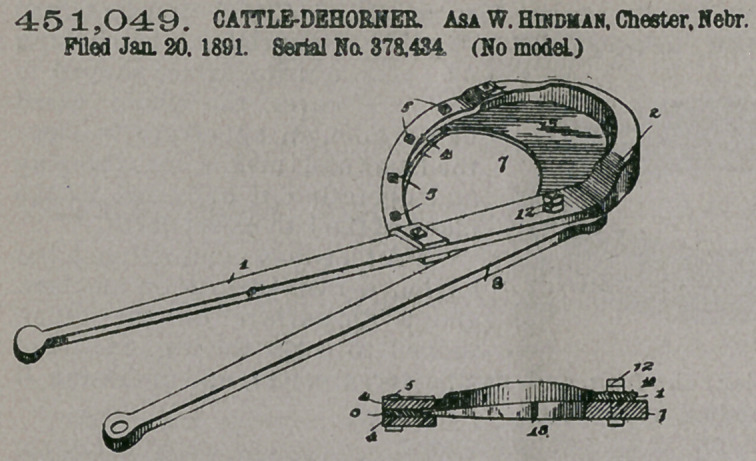


**Figure f7:**
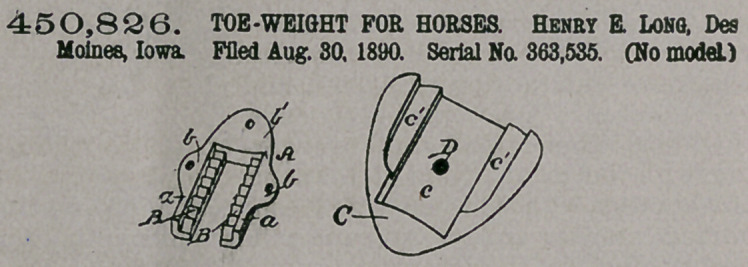


**Figure f8:**
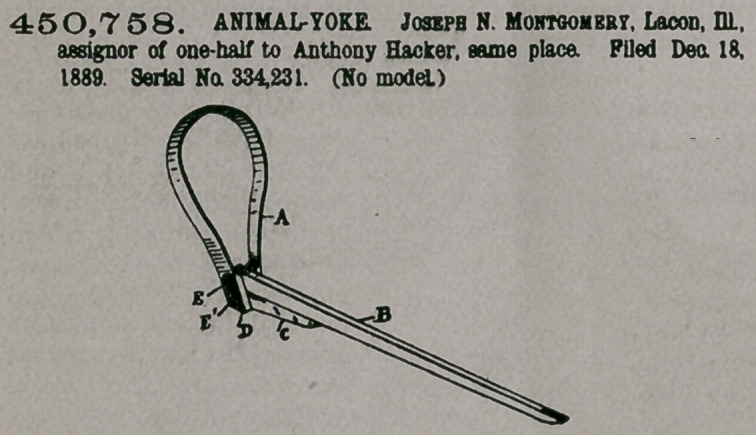


**Figure f9:**
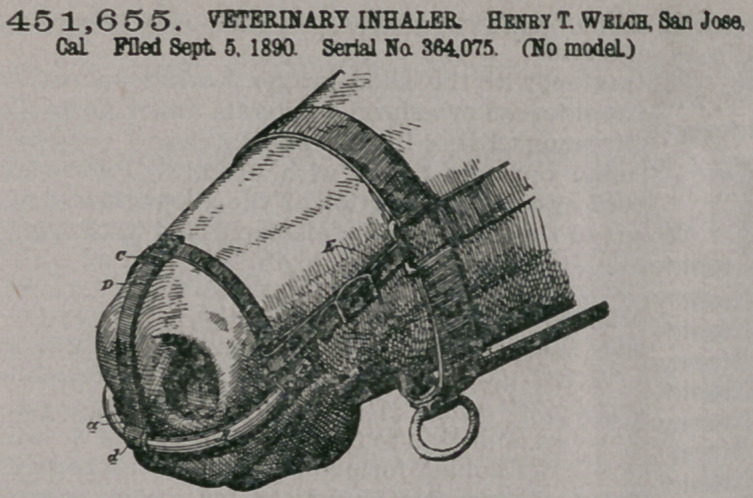


**Figure f10:**
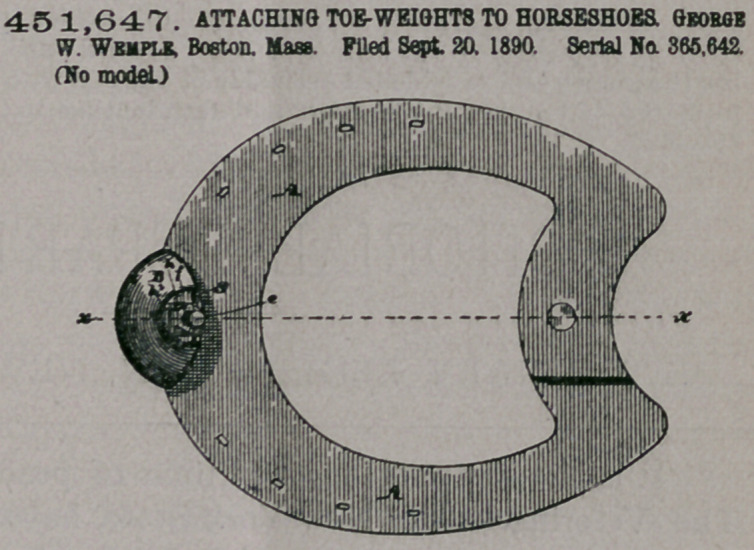


**Figure f11:**
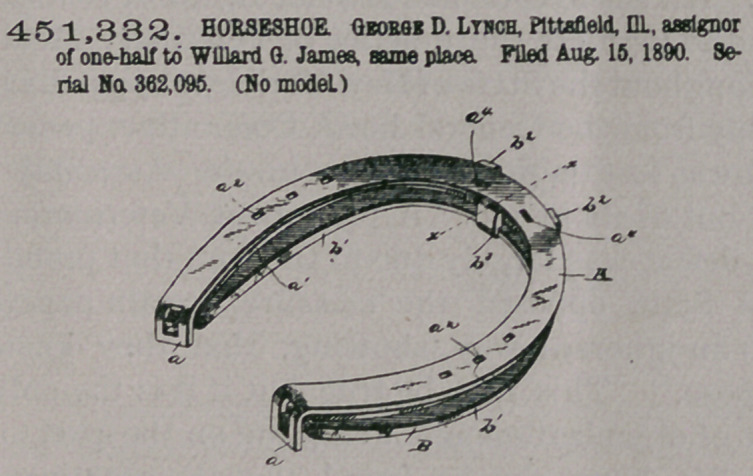


**Figure f12:**